# Predicting different adhesive regimens of circulating particles at blood capillary walls

**DOI:** 10.1007/s10404-017-2003-7

**Published:** 2017-10-26

**Authors:** A. Coclite, H. Mollica, S. Ranaldo, G. Pascazio, M. D. de Tullio, P. Decuzzi

**Affiliations:** 1 Laboratory of Nanotechnology for Precision Medicine, nPMed, Fondazione Istituto Italiano di Tecnologia, Via Morego 30, 16163 Genoa, Italy; 2 Centro di Eccellenza in Meccanica Computazionale, CEMeC, Politecnico di Bari, Via Re David, 200, 70125 Bari, Italy; 3 Dipartimento di Meccanica, Matematica e Management, DMMM, Politecnico di Bari, Via Re David, 200, 70125 Bari, Italy

**Keywords:** Drug delivery, Lattice Boltzmann, Immersed boundary, Computational modeling, Computational nanomedicine

## Abstract

A fundamental step in the rational design of vascular targeted particles is the firm adhesion at the blood vessel walls. Here, a combined lattice Boltzmann–immersed boundary model is presented for predicting the near-wall dynamics of circulating particles. A moving least squares algorithm is used to reconstruct the forcing term accounting for the immersed particle, whereas ligand-receptor binding at the particle–wall interface is described via forward and reverse probability distributions. First, it is demonstrated that the model predicts with good accuracy the rolling velocity of tumor cells over an endothelial layer in a microfluidic channel. Then, particle–wall interactions are systematically analyzed in terms of particle geometries (circular, elliptical with aspect ratios 2 and 3), surface ligand densities (0.3, 0.5, 0.7 and 0.9), ligand-receptor bond strengths (1 and 2) and Reynolds numbers (*Re* = 0.01, 0.1 and 1.0). Depending on these conditions, four different particle–wall interaction regimens are identified, namely not adhering, rolling, sliding and firmly adhering particles. The proposed computational strategy can be efficiently used for predicting the near-wall dynamics of particles with arbitrary geometries and surface properties and represents a fundamental tool in the rational design of particles for the specific delivery of therapeutic and imaging agents.

**Electronic supplementary material:**

The online version of this article (doi:10.1007/s10404-017-2003-7) contains supplementary material, which is available to authorized users.

## Introduction

A plethora of nano-/microparticles have been developed over the last decade for the precise delivery of therapeutic and imaging agents in the treatment and early detection of a variety of diseases, including cancer and cardiovascular (Peer et al. 2007; Mulder et al. 2014). Over free drug molecules and contrast agents, systemically injectable particles offer multiple advantages, such as improved organ biodistribution, enhanced accumulation at diseased sites and protection of the therapeutic cargo from a rapid enzymatic degradation (Bao et al. 2013; Min et al. 2015; Muthu et al. 2014). Top-down fabrication approaches have been proposed for precisely and independently tailoring the size, shape, surface properties and, more recently, the mechanical stiffness of particles—the so-called 4S parameters in the rational design of particles (Key et al. 2013, 2015; Euliss et al. 2006; Anselmo and Mitragotri 2016). Specifically, the particle size may vary from a few tens of nanometers to few a microns; the shape can be spherical, discoidal, cylindrical and spheroidal; the surface can be decorated with a variety of ligand molecules for specific cell recognition; and the particle structure can be soft as cells or stiff as metals. The ability to finely tune the 4S parameters allows us, on the one hand, to fabricate particles with a large variety of configurations, de facto enabling rational particle design, but, on the other hand, requires sophisticated computational tools for wisely selecting optimal particle configurations, depending on the biological target. Indeed, given the number of possible combinations, a rational selection solely based on experimental testing is practically unfeasible.

Moved by this need, in recent years, the authors and other scientists have started developing and employing new mathematical and computational tools for predicting the vascular and extravascular behavior of particles in terms of the 4S parameters (Decuzzi 2016). For instance, at the macro-vascular scale, the isogeometric analysis (IA) was fruitfully exploited to predict the vascular deposition of microparticles, directly infused via a catheter positioned within the left coronary artery, as a function of the endothelial receptor densities (Hossain et al. 2013, 2014). Similarly, direct numerical simulations (DNS) and the immersed boundary (IB) were employed to predict the fluid–structure interaction of bodies with arbitrary shapes immersed in an incompressible fluid (Coclite et al. 2016; de Tullio and Pascazio 2016). At the microscopic scale, the immersed finite element method (IFEM) was also used to study the transport of micro- and nanoparticles within whole blood and demonstrate that sub-micro- and micron-sized particles would tend to be pushed laterally toward the vessel walls by the fast-moving and more abundant red blood cells (Lee et al. 2013, 2014).

More recently, the lattice Boltzmann (LB) method was also employed to solve transport problems of biological relevance. Because of its simple implementation and high parallel performance, LB is a suitable method for describing complex flow behaviors across a wide range of length and temporal scales (Succi 2001, 2008; Aidun and Clausen 2010). This computational tool was efficiently applied to follow the dynamics of rigid particles and deformable capsules, such us red blood cells (RBCs) and leukocytes, in whole blood capillary flow. Specifically, it was applied to finely tune the geometry and viscoelastic properties of RBCs in order to accurately replicate the rheological response of whole blood as well as to reduce computing burden (Fedosov et al. 2010; Sun and Munn 2005; Kruger et al. 2011), predict the clustering of RBCs and microcapsules in narrow capillaries (McWhirter et al. 2009), explain the role of RBCs on the vascular rolling of leukocytes (Sun et al. 2003), determine numerically the size of the cell-free layer developing next to the vessel walls (Fedosov et al. 2010) and model the vascular transport of micro-/nanoparticles (Coclite et al. 2016; Gekle 2016; Tan et al. 2016; Basagaoglu et al. 2013).

In this work, a LB–IB method is further developed for predicting the adhesive interaction of particles with blood vessel walls, under capillary flow. The particle surface is decorated with ligand molecules, mediating specific adhesive interaction with counter-molecules (receptors) distributed over the vessel walls. These interfacial molecular adhesive forces are computed through a probabilistic approach determining bond formation and destruction over the entire particle surface (Sun and Munn 2008). The near-wall dynamics of circular and elliptical particles, with two aspect ratios, is analyzed at three Reynolds numbers (*Re* = 0.01, 0.1, 1.0), for three different densities of the surface ligands and two different values of the ligand-receptor chemical affinity. A direct comparison between computational predictions and experimental measurements is also presented in the case of rolling tumor cells on vascular endothelium in a microfluidic chip for assessing the model accuracy. Then, particle–wall interaction maps are derived in terms of particle shape, ligand density, bond strength and flow conditions.

## Computational method

The mathematical method used to model the fluid evolution and the fluid–structure interaction, proposed and validated by Coclite et al. (2016), is briefly described in the following.

### The combined lattice Boltzmann–immersed boundary (LB–IB) method

The evolution of the fluid is defined in terms of a set of *N* discrete distribution functions $\left\{ {f_{i} } \right\}$ (*i* = 0, …, *N* − 1) which obey the dimensionless Boltzmann equation

1 $$.f_{i} \left( {\varvec{x} + \varvec{e}_{\varvec{i}}\Delta t,t +\Delta t} \right) - f_{i} \left( {\varvec{x},t} \right) = - \frac{{\Delta t}}{\tau }\left[ {f_{i} \left( {\varvec{x},t} \right) - f_{i}^{\text{eq}} \left( {\varvec{x},t} \right)} \right],$$

in which ***x*** and *t* are the spatial and time coordinates, respectively, [***e***_***i***_] (*i* = 0, …, *N* − 1) is the set of discrete velocities, ∆*t* is the time step, and *τ* is the relaxation time given by the unique non-null eigenvalue of the collision term in the BGK-approximation (Bhatnagar et al. 1954). The kinematic viscosity of the flow is related to the single relaxation time *τ* as $\upsilon = c_{s}^{2} \left({\tau - \frac{1}{2}} \right){\Delta}t$ being $c_{s} = \frac{1}{\sqrt 3 }\frac{{\Delta x}}{{\Delta t}}$ the reticular speed of sound. The moments of the distribution functions define the fluid density $\rho = \mathop \sum \nolimits_{i} f_{i}$, velocity $\varvec{u} = \mathop \sum \nolimits_{i} f_{i} \varvec{e}_{\varvec{i}} /\rho$ and the pressure $p = c_{s}^{2} \rho = c_{s}^{2} \mathop \sum \nolimits_{i} f_{i}$. The local equilibrium density functions [$f_{i}^{\text{eq}}$] (*i* = 0, …, *N* − 1) are expressed by the Maxwell–Boltzmann (MB) distribution,

2 $$f_{i}^{\text{eq}} \left( {\varvec{x},t} \right) = \omega_{i} \rho \left[ {1 + \frac{1}{{c_{s}^{2} }} \left( {\varvec{e}_{\varvec{i}} \cdot \varvec{u}} \right) + \frac{1}{{2c_{s}^{4} }} \left( {\varvec{e}_{\varvec{i}} \cdot \varvec{u}} \right)^{2} - \frac{1}{{2c_{s}^{2} }} \varvec{u}^{2} } \right].$$

On the two-dimensional square lattice with *N* = 9 speeds (D2Q9) (Qian et al. 1992), the set of discrete velocities is given by:

3 $$\varvec{e}_{\varvec{i}} = \left\{ {\begin{array}{*{20}l} {\left( {0,0} \right),} \hfill & {{\text{if}}\; i = 0} \hfill \\ {\left( {\cos \left( {\frac{{\left( {i - 1} \right)\pi }}{2}} \right), \sin \left( {\frac{{\left( {i - 1} \right)\pi }}{2}} \right)} \right),} \hfill & {{\text{if}}\; i = 1 - 4} \hfill \\ {\sqrt 2 \left( {\cos \left( {\frac{{\left( {2i - 9} \right)\pi }}{4}} \right), \sin \left( {\frac{{\left( {2i - 9} \right)\pi }}{4}} \right)} \right),} \hfill & {{\text{if}}\; i = 5 - 8} \hfill \\ \end{array} } \right.,$$

with the weight, *ω*_*i*_ = 1/9 for *i* = 1–4, *ω*_*i*_ = 1/36 for *i* = 5–8, and *ω*_0_ = 4/9. Here, we adopt a discretization in the velocity space of the MB distribution based on the Hermite polynomial expansion of this distribution (Shan et al. 2006).

An effective forcing term accounting for the boundary presence, ${\mathcal{F}}_{i}$, can be included as an additional factor on the right-hand side of Eq. (11).

Following the argument from Guo et al. (2002), also developed in (De Rosis et al. 2014a, b; Suzuki et al. 2015; Wang et al. 2015), ${\mathcal{F}}_{i}$ is given by:

4 $${\mathcal{F}}_{i} = \left( {1 - \frac{1}{2\tau }} \right)\omega_{i} \left[ {\frac{{\varvec{e}_{i} - \varvec{u}}}{{c_{s}^{2} }} + \frac{{\varvec{e}_{i} \cdot \varvec{u}}}{{c_{s}^{4} }}\varvec{e}_{i} } \right] \cdot \varvec{f}_{ib} ,$$

where ***f***_*ib*_ is the body force term evaluated through the formulation by Favier et al. (2014), combined with the moving least squares reconstruction (Vanella and Balaras 2009) in the immersed boundary technique by Coclite et al. (2016). Due to the presence of the forcing term ${\mathcal{F}}_{i}$, the macroscopic quantities, given by the moments of the distribution functions, are obtained as:

5 $$\rho = \mathop \sum \limits_{i} f_{i} ,$$

6 $$\rho \varvec{u} = \mathop \sum \limits_{i} f_{i} \varvec{e}_{\varvec{i}} + \frac{{\Delta \varvec{t}}}{2}{\mathbf{\mathcal{F}}}_{\varvec{i}} ,$$

It is proved that in such a framework one can recover the forced Navier–Stokes equations with second-order accuracy (Guo et al. 2002; Favier et al. 2014). In the present model, the forcing term accounts for the presence of an arbitrary-shaped body into the flow field, whereas the external boundaries of the computational domain are treated with the known-velocity bounce back conditions by Zou and He (1997).

### Pressure and viscous stresses

Let *nl* be the number of linear elements composing the surface of the immersed body being $l$ the element index; the pressure and viscous stresses exerted by the immersed body are:

7 $$\varvec{F}_{p} \left( t \right) = \mathop \sum \limits_{l = 1}^{nl} \left( { - p_{l} \varvec{n}_{\varvec{l}} } \right)S_{l} ,$$

8 $$\varvec{F}_{p} \left( t \right) = \mathop \sum \limits_{l = 1}^{nl} \left( {\bar{\tau }_{l} \cdot \varvec{n}_{\varvec{l}} } \right)S_{l}$$

where $\bar{\tau }_{l}$ and $p_{l}$ are the viscous stress tensor and the pressure evaluated in the centroid of the $l$th element, respectively; $\varvec{n}_{l}$ is the outward normal unit vector, while $S_{l}$ is the length of the $l$th element. The pressure and velocity derivatives in Eqs. (7) and (8) are evaluated considering a probe in the normal positive direction of each element, the probe length being $1.2\Delta x$, and using the moving least squares formulation cited (Coclite et al. 2016). In this framework, the velocity derivatives evaluated at the probe are considered equal to the ones on the linear element centroid as previously done by the authors (Coclite et al. 2016; de Tullio and Pascazio 2016).

### Wall–particle interaction

The adhesion model used in the present work is based on the works by Sun et al. (2003, 2008). Ligand and receptor molecules are distributed over the particle and vessel wall surfaces, respectively. Ligand molecules are modeled as linear springs which, by interacting with wall receptor molecules, tend to establish bonds (ligand-receptor bonds) and support a mechanical force $f_{l,b}$ given as:

9 $$\varvec{f}_{l,b} = \sigma \left( {y_{l} - y_{\text{cr,eq}} } \right)\varvec{n}_{l} ,$$

with $y_{l}$ the bond length, $y_{\text{cr,eq}}$ the equilibrium bond length and $\sigma$ the spring constant. The receptor density is assumed uniform; the solid wall is supposed completely covered by receptive molecules. In the present model, all springs have the same spring constant, $\sigma$. The total adhesive force, $\varvec{F}_{b}$, is obtained by integrating $\varvec{f}_{l,b}$ over the particle perimeter. Bonds can be only generated if the minimum separation distance between the particle boundary and the wall is smaller than a critical value, $y_{\text{cr}} = 6.8 \times 10^{ - 3} \,H$. The equilibrium bond length, resulting in a null force, is chosen as $y_{\text{cr,eq}} = 0.5 y_{\text{cr}}$. All lengths are normalized by the channel height, *H*. The linear spring constant is computed in *lattice units* and non-dimensionalized through the term $\frac{{\varrho_{\text{ref}} \upsilon^{2} }}{H}$, where $\rho_{\text{ref}}$, $H$ and $\upsilon$ are reference density, length and kinematic viscosity, respectively.

The bond formation is regulated by a forward probability function

10 $$P_{f} = 1 - { \exp }\left( { - k_{f} Nl\Delta t} \right),$$

with $k_{f}$ forward bond rate and $Nl$ the number of ligand actually probing the surface (number of *active* elements) over the total number of linear elements. At each time step, a pre-existing bond can be destroyed according to the reverse probability function

11 $$P_{r} = 1 - \exp \left({- k_{r0} \exp \left({\frac{{\left({\sigma - \sigma^{*}} \right)\left({y_{l} - y_{\text{cr,eq}}} \right)^{2}}}{{2k_{\text{B}} T}}} \right)\Delta t} \right).$$

Here $k_{r0}$ is the reverse bond rate, $\sigma^{*}$ is the equilibrium spring constant (taken as $0.5 \sigma$), and $k_{\text{B}} T$ is the thermal potential (Sun et al. 2003; Sun and Munn 2008). The equilibrium spring constant $\sigma^{*}$ enables to model two different classes of ligand-receptor bonds: ‘slip’ bonds for $\sigma > \sigma^{*}$, where forces exerted on the bond facilitate disentanglement, and ‘catch’ bonds for $\sigma < \sigma^{*}$, where forces exerted on the bond facilitate entanglement. Here, by fixing $\sigma^{*} = 0.5\sigma$, slip bonds are considered which are far more common in the case of leukocyte and cancer cell rolling/adhesion (Marshall et al. 2003).

A Van der Waals-like potential is implemented to model the particle–wall interaction. The force **F**_w_ is so applied along the solid walls positive normal directions into the particle centroid (Sun et al. 2003)

12 $$\varvec{F}_{w} = \frac{{H_{k} }}{8\sqrt 2 }\sqrt {\frac{r}{{ \epsilon^{5} }}} \varvec{n},$$

being $\varvec{n}$ the solid wall normal direction unit vector, $H_{k}$ the Hamaker constant, $r$ the particle radius and $\epsilon$ the separation distance between the particle and the wall. The Hamaker constant is non-dimensionalized through the term $\rho_{\text{ref}} H\upsilon^{2}$.

### Fluid–structure interaction strategy

The total force ***F***(*t*) and total moment ***M***(*t*) acting on the immersed body are evaluated in time, and the translation and rotation of the particle are updated at each Newtonian dynamics time step by an explicit second-order scheme. Therefore, the linear and angular accelerations are obtained directly as

13 $$\dot{\varvec{u}}\left( t \right) = \varvec{F}\left( t \right)/m,$$

14 $$\dot{\omega }\left( t \right) = \varvec{M}\left( t \right)/I,$$

being *m* and *I* the particle mass and inertia moment of the two-dimensional particle about its centroid, respectively. The linear and angular velocities are computed as

15 $$\varvec{u}\left( t \right) = \frac{2}{3}\left( {2\varvec{u}\left( {t -\Delta t} \right) - \frac{1}{2}\varvec{u}\left( {t - 2\Delta t} \right) + \dot{\varvec{u}}\left( t \right)\Delta t} \right),$$

16 $$\omega \left( t \right) = \frac{2}{3}\left( {2\omega \left( {t -\Delta t} \right) - \frac{1}{2}\omega \left( {t - 2\Delta t} \right) + \dot{\omega }\left( t \right)\Delta t} \right),$$

with $\Delta x =\Delta t = 1$. Here, a weak coupling approach between the fluid and the particle is implemented. Note that this approach is unconditionally stable for small velocity variations (Zhang and Hisada 2004), which is indeed the case of the present work.

## Results

### Cell rolling in a capillary flow

**Fig. 1:**
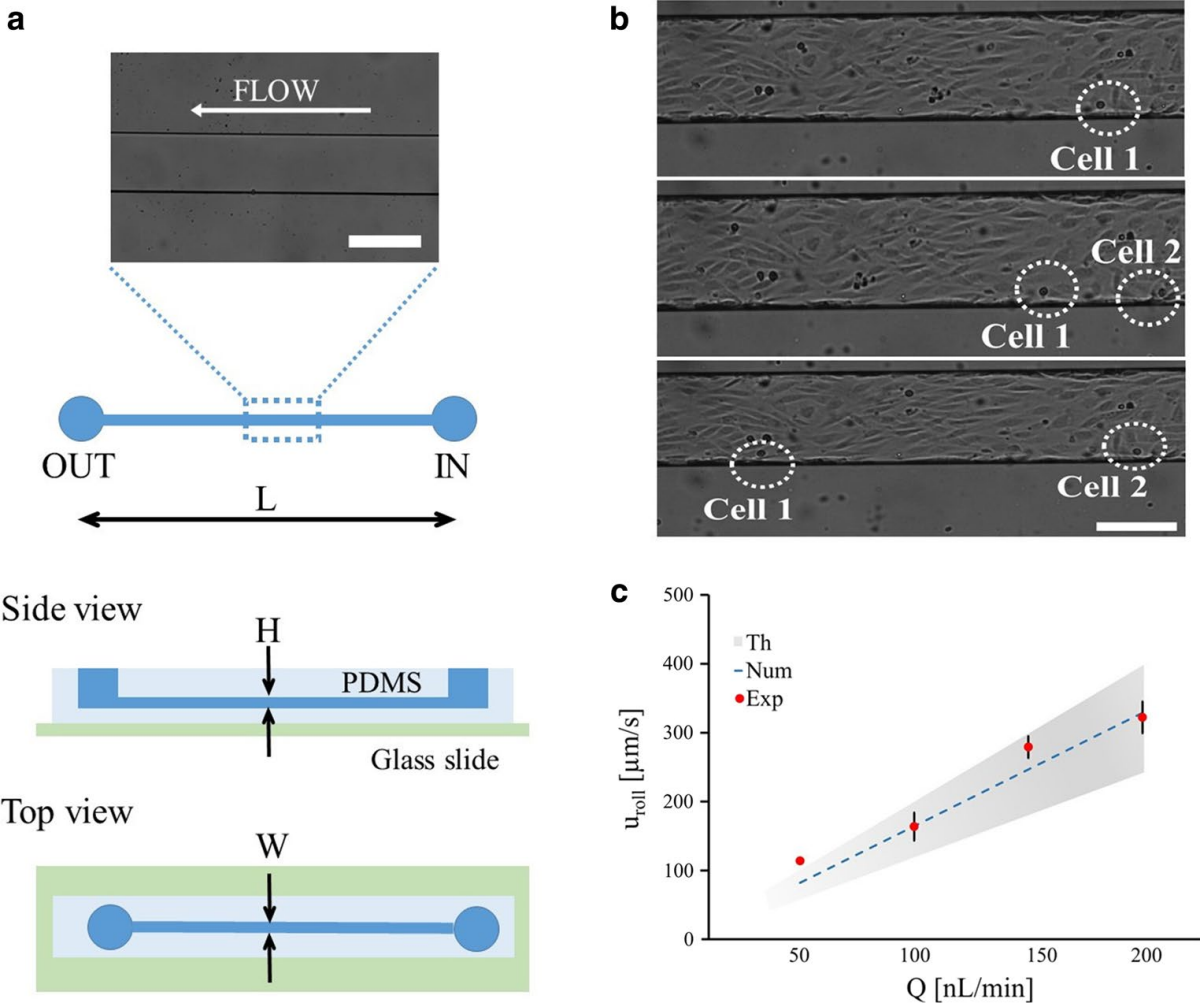
HCT-15 cells rolling on an HUVEC monolayer into a single-channel microfluidic chip. **a** Schematic representation of the single-channel microfluidic chip with definition of the main geometric quantities. From top to bottom: bright field epi-fluorescent microscope image of the region of interests (scale bar 250 μm); side and top views of the chip (*L* = 2.7 cm, *H* = 42 μm, *W* = 210 μm). **b** Representative images of HCT-15 cells rolling over a confluent monolayer of HUVECs (×10 magnification, scale bar 250 μm). **c** Rolling velocity of HCT-15 under four different flow conditions (50, 100, 150 and 200 nL/min) estimated via numerical and theoretical analyses

To reproduce typical capillary flow conditions, a single-channel microfluidic chip is realized in PDMS, following standard fabrication procedures (Fig. 1a) (Manneschi et al. 2016). First, a negative mold of the channel is generated, upon UV light cross-linking, baking and development of a SU-8 film. Then, a PDMS replica of the mold is realized and peeled off after curing. Two circular holes of ~ 1 mm are punched into the PDMS layer, constituting the inlet and outlet of the microfluidic chip. Finally, following an oxygen plasma treatment, the PDMS layer is bonded to a glass slide. The resulting microfluidic chip has a channel with width *W* = 210 µm, height *H* = 42 µm and length *L* = 2.70 cm. Top and side views as well as an optical microscopy image of the chip are presented in Fig. 1a.

To establish a confluent cell layer resembling the microvascular endothelium, human umbilical vein endothelial cells (HUVECs) are cultured within the channel. Specifically, after autoclaving the chip in DI water for 2 h at 120 °C, the channel is first filled with fibronectin (20 µg/mL) and then with HUVECs (2 × 10^6^ cells/mL). Then, the chip is kept in an incubator for about 2 days, until a confluent monolayer of HUVECs is established. At this point, 10^6^ colon rectal tumor cells (HCT-15) are injected in the endothelialized channel via a syringe pump at different flow rates, namely *Q* = 50, 100, 150 and 200 nL/min. The vascular transport of HCT-15 cells is monitored for about 15 min using a fluorescent microscope, and the rolling velocity is estimated within the region of interest. Note that the considered flow rates *Q* correspond to physiological and tumor characteristic wall shear rates *S*, namely *S* = 13.5, 27, 40.5 and 54 s^−1^ $(S = 6Q/(WH^{2} ))$. Also, the corresponding mean flow velocities U are 94.48, 188.9, 283.4 and 377.9 μm/s $(U = Q/(WH))$, respectively.

Still images of cells rolling over the endothelial monolayer within the chamber are presented in Fig. 1b, at different time points. The rolling velocity u_r_ is defined as the ratio between the distance traveled by the cell and the period of observation and is derived by post-processing the fluorescent microscopy images. As shown in Fig. 1c, the rolling velocity (red dots) increases linearly as the flow rate *Q* grows (*R* ^2^ = 0.966), ranging from 113.9 ± 4.13 for 50 nL/min to 322.2 ± 22.8 for 200 nL/min. This was expected and confirmed by theoretical and numerical predictions.

The theoretical rolling velocity *u*_th_ is derived assuming the cell as a rigid sphere of diameter d rolling in a rectangular channel pushed by a flow with rate *Q*, as derived for a channel of rectangular cross section (Bird 2002), so that

17 $$u_{\text{th}} = \frac{3}{2}\frac{Q}{WH}\left[ {1 - \left( {1 - \frac{d}{H}} \right)^{2} } \right].$$

Considering that the diameter of HCT-15 cells ranges between 14 and 20 μm (*d* = 15 ± 3 μm), it results in *u*_th_ = 83.15 ± 19.7, 166.1 ± 39.4, 249.0 ± 59.1 and 333.0 ± 78.7 μm/s, for each of the four considered flow rates. This rolling velocity is also estimated using the present LB–IB model assuming the cell as a circular particle, settled at a distance 3 × 10^−3^ H from the wall and with a ligand density 0.3. The assumed ligand density value of 0.3 returns a good agreement between the experimental and numerical predictions for the cell rolling velocity over four different flow rates. The ligand density represents the ratio between the number of ligand molecules on the circulating cell and the number of receptor molecules distributed over the endothelial cells lining the blood vessel walls. As expected, both the numerical and theoretical rolling velocities vary linearly with *Q* and are in very good agreement with each other (*R* ^2^ = 0.994). The experimental, theoretical and numerical rolling velocities are all plotted in Fig. 1c, for the four flow rates *Q*. Given the variation in cell diameter, shadowed areas are used to present the rolling velocities, whose upper and lower limits are associated with the bigger and smaller cell diameters, respectively. The present LB–IB model accurately predicts the rolling velocity of tumor cells over a wide range of flow rates.

### Modeling the adhesion dynamics of near-wall circulating particles

In this section, the adhesive dynamics of particles circulating in close proximity of the blood vessel walls is predicted employing the present LB–IB computational approach. Vascular adhesion is assessed in terms of physiological parameters, such as the local hemodynamic conditions—the Reynolds number $\left( {Re = \frac{{H\,u_{max} }}{{\upsilon_{\text{ref}} }}} \right)$, based on the upper wall velocity, $u_{max}$, the capillary height, *H*, and the reference kinematic viscosity, $\upsilon_{\text{ref}}$; particle parameters such as the particle shape—circular and elliptical; and density of ligand molecules (*ρ*_l_) decorating the particle perimeter.

**Fig. 2:**
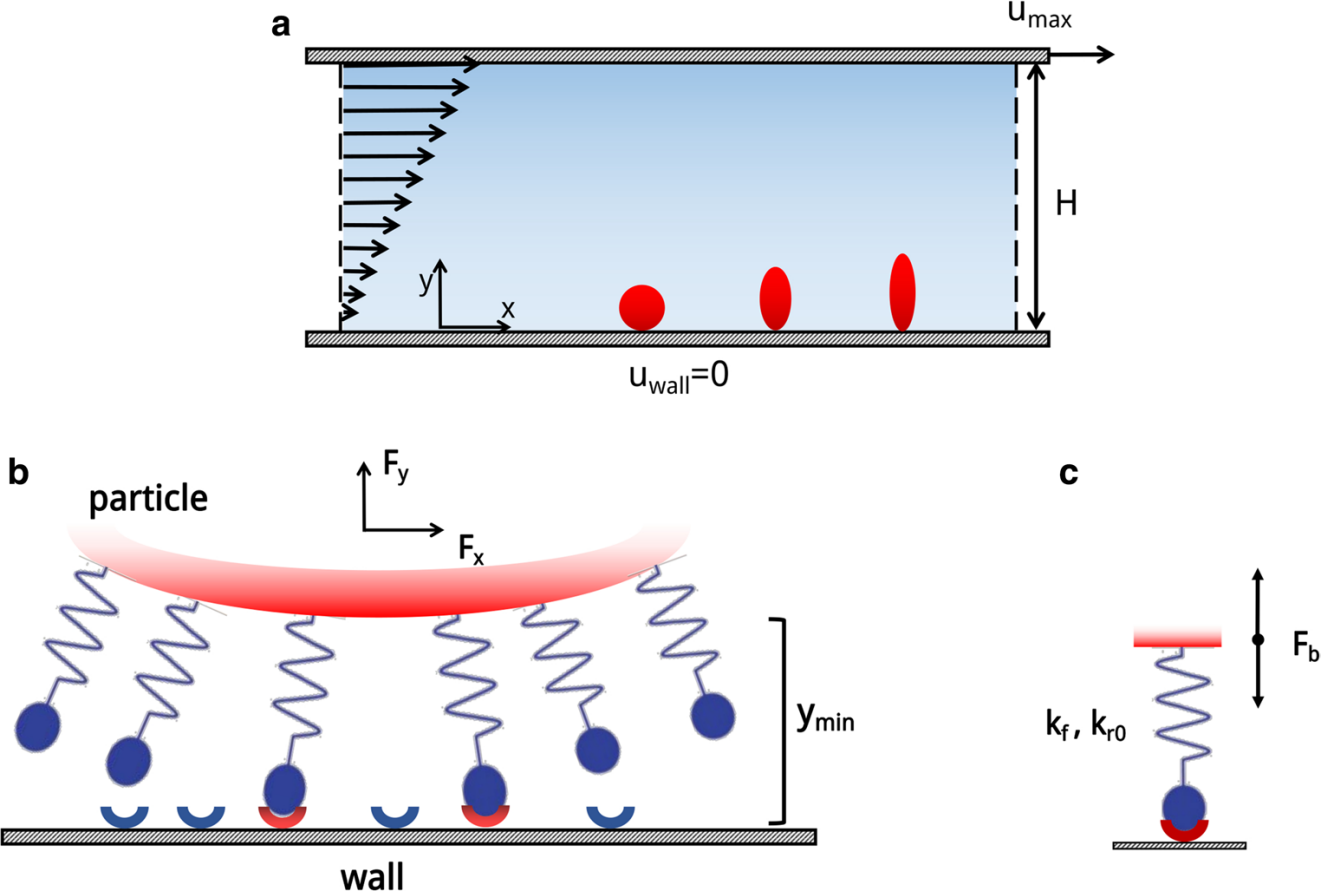
Particle transport in a linear laminar flow. **a** Schematic representation of the computational domain. **b** Ligand distributed over the particle perimeter interacting with receptors distributed over the vessel wall. **c** Ligand-receptor bond modeled as a spring with characteristic forward $k_{f}$ and reverse $k_{r0}$ strengths

The computational domain resembles the near-wall region in a capillary flow and is limited at the bottom (*y* = 0) by a fixed wall (the vessel wall in a blood capillary) and at the top (*y* = *H*) by a moving wall (interface between the cell-free layer and the core of the blood capillary) (Fig. 2a). Within this domain, a linear shear rate is imposed and the upper wall has a velocity *u*_max_. The height *H* of the computational domain coincides with the height of the microfluidic chip used before for the experimental validation. The computational domain is confined within the area $\left[ {0,10H\left] \times \right[0,H} \right]$, where *H* is discretized with 200 points. Periodic boundary conditions are imposed on the two sides (*x* = 0 and *x* = 10*H*); zero slip velocities are imposed at the bottom (*u* = 0) and top (*u* = u_max_) walls so that the linear flow field follows the relationship $u_{x} \left( {0,y} \right) = u_{max} y/H = \left( {Re \cdot \upsilon_{\text{ref}} /H} \right)y/H$.

**Fig. 3:**
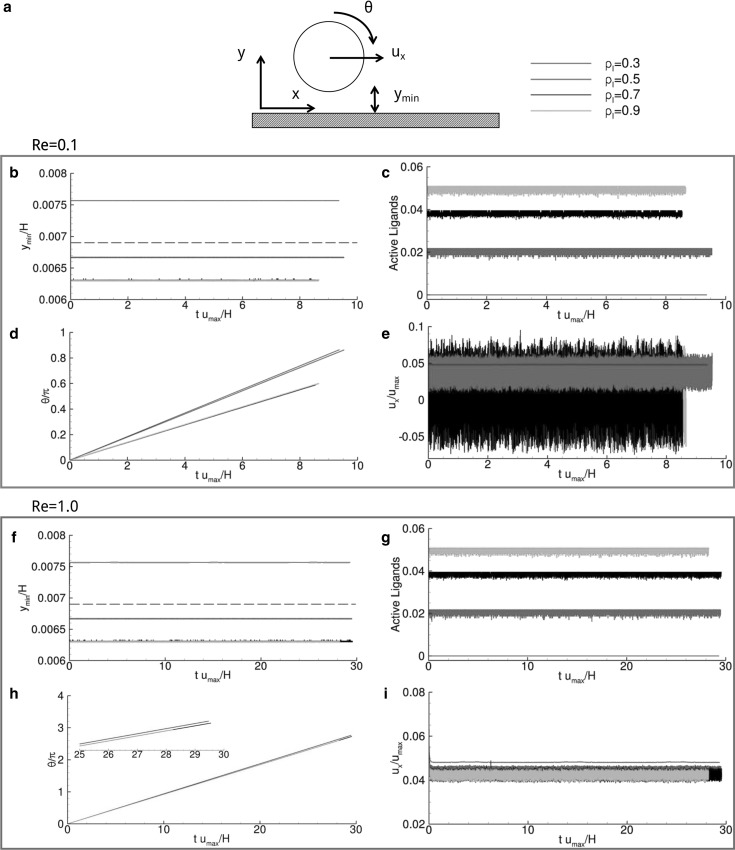
Vascular adhesion of circular particles ($\sigma = 2$). **a** Schematic representation of the problem. **b**, **f** Particle separation distance from the wall versus time. The dashed line corresponds to $y_{\text{cr}}$. **c**, **g** Active over total number of ligands versus time. **d**, **h** Angular rotation, $\theta,$ versus time where the inset presents a magnified view within the interval $25 \le tu_{max} \le 30$. **e**, **i** Normalized rolling velocity versus time

Particles, initially at rest, are placed in the computational domain at a separation distance from the bottom wall equal to *y*_0_ = 3×10^−3^ H. Three different geometries are considered for the particles, namely circular, elliptical with an aspect ratio 2 and elliptical with an aspect ratio 3 (Fig. 3a). The characteristic size is chosen as to keep constant the total area enclosed by the particle, namely *A* = 0.025 *H* ^2^. Thus, the circular particle has a diameter of 0.18 *H*, and the elliptical particles have axial lengths equal to 0.25 *H* × 0.125 *H* and 0.31 *H* × 0.103 *H*, respectively. On the particle perimeter, ligand molecules are uniformly distributed with a density *ρ*_l_ of 0.3, 0.5, 0.7 and 0.9 (Fig. 2b, c). On the vessel walls, a receptor density equal to 1 is imposed. The ligand-receptor bonds are characterized by an adhesive bond strength σ and a biochemical affinity *k*_*f*_/*k*_*r*,0_ = 8.5 × 10^3^. All parameters used in the model are listed in Table 1, with their dimensional and non-dimensional values, and the schematic representations of the computational domain and particles are given in Fig. 2.

**Table 1:**
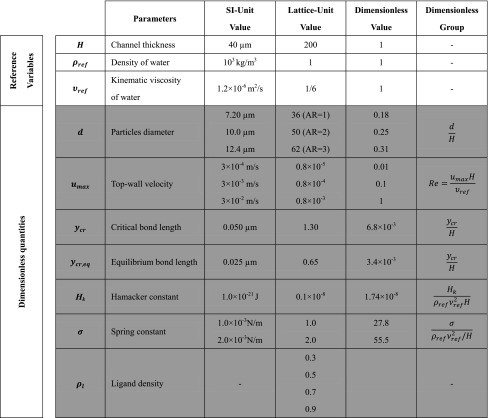
Parameters used in the computational experiments expressed in the SI unit system and in lattice unit system along with their dimensionless groups

Note that, the channel thickness, *H*, the water density, $\rho_{\text{ref}}$, the water kinematic viscosity, $\upsilon_{\text{ref}}$, are used throughout the formulation to present in dimensionless form all other dependent physical quantities, while all quantities for the description of the physical problem are shaded

### Vascular adhesion dynamics for circular particles

Data on the adhesive dynamics of circular particles are shown in Fig. 3 and listed in Table 2, for *Re* = 0.01, 0.1 and 1.0. A circular particle, initially settled in close proximity of the vessel wall (*y*_0_ = 3.0×10^−3^ H) (Fig. 3a), rapidly forms ligand-receptor bonds initiating the adhesion process. Note that the initial separation distance *y*_0_ is smaller than the critical distance for bond formation (*y*_cr_ = 6.8×10^−3^ H; dashed lines in Fig. 3).

**Table 2:** Circular particle kinematics and dynamics quantities obtained for *σ* = 2

Reynolds number		Ligand density			
		0.3	0.5	0.7	0.9
0.01	*y*_min_/*H*	7.57 × 10^−3^	6.0 × 10^−3^	6.0 × 10^−3^	6.0 × 10^−3^
	*u*_roll_/*u*_max_	4.43 × 10^−2^	0	0	0
	Ω*H*/*u*_max_	0.280	0	0	0
0.1	*y*_min_/*H*	7,57 × 10^−3^	6.67 × 10^−3^	6.29 × 10^−3^	6.29 × 10^−3^
	*u*_roll_/*u*_max_	4.80 × 10^−2^	4.08 × 10^−2^	0.69 × 10^−2^	0.68 × 10^−2^
	Ω*H*/*u*_max_	0.29	0.25	0.125	0.125
1	*y*_min_/*H*	7,57 × 10^−3^	6.67 × 10^−3^	6.29 × 10^−3^	6.29 × 10^−3^
	*u*_roll_/*u*_max_	4.80 × 10^−2^	4.55 × 10^−2^	4.20 × 10^−2^	4.20 × 10^−2^
	Ω*H*/*u*_max_	0.29	0.284	0.280	0.280

Separation distance, *y*_min_/*H*, rolling velocity, *u*_roll_/*u*_max_, and rotational velocity, $\Omega H/u_{max}$, are tabulated as function of the Reynolds number and the density of ligands ($\rho_{l}$)

The equilibrium position of the particle with respect to the wall is given by *y*_min_, the minimum separation distance, which is plotted in Fig. 3b, f, respectively, for *Re* = 0.1 and 1.0. The particle with a ligand density *ρ*_l_ = 0.3 moves away from the wall returning an equilibrium separation distance *y*_min_ = 7.6×10^−3^ H, which is larger than the critical distance for bond formation. Thus, for *ρ*_l_ = 0.3 and smaller, the ligand density is insufficient to induce the formation of any stable bonds and the particle moves away from the wall—*not adhering particle*. For larger ligand densities, the separation distance at equilibrium reduces returning values of 6.67 × 10^−3^ H, 6.29 × 10^−3^ H and 6.3 × 10^−3^ H, for *ρ*_l_ = 0.5, 0.7 and 0.9, respectively. These are all cases where the equilibrium position is smaller than the critical bond distance *y*_cr_, and stable ligand-receptor bonds are formed. Indeed, as *ρ*_l_ increases, the hydrodynamic forces exerted over the particle are redistributed over a larger number of ligands, thus diminishing the deformation of each ligand-receptor bonds and moving the particle closer to the wall (Fig. 3b, f).

As documented in Fig. 3c, g, the percentage of active ligands increases with ρ_l_; in other words, the number of closed ligand-receptor bonds grows with the number of ligands decorating the particle perimeter. Note that, since ligand-receptor binding is defined in a statistical manner, the number of bonds oscillates over time around an average value. For *ρ*_l_ = 0.3, the number of active bonds is zero (*not adhering particle*), whereas it grows to 0.020, 0.036 and 0.048, for *ρ*_l_ = 0.5, 0.7 and 0.9, respectively. Oscillations in the number of active ligands appear as bands in Fig. 3c, g. It is here important to highlight that, due to the small region of contact between a circular particle and the wall, only a small number of ligand-receptor bonds are formed even in the case of high ligand densities. As from Fig. 3c, g, the percentage of active ligands is equal to 2% for *ρ*_l_ = 0.5 and grows only up to ~ 5% for *ρ*_l_ = 0.9.

The kinematic parameters, namely the angular rotation *θ* and longitudinal velocity *u*_*x*_ of the particle, are presented in the remaining insets of Fig. 3. The variation of *θ* over time is given in Fig. 3d, h. It shows a steady and linear increase in θ, thus implying a constant angular velocity Ω of the particle over the wall—*rotating*, *not adhering particle*. The rotational velocity reduces as the number of ligand-receptor bonds increases and is equal to Ω*H*/*u*_max_ = 0.29, 0.25, 0.125 and 0.125, respectively, for *ρ*_l_ = 0.3, 0.5, 0.7 and 0.9, at *Re* = 0.1. For larger Reynolds numbers (*Re* = 1.0), the angular velocity *Ω* exhibits a negligible variation with *ρ*_l_, possibly because of the larger hydrodynamic dislodging forces. In this condition, Ω*H*/*u*_max_ is equal to 0.29, 0.284, 0.280 and 0.280.

Finally, the normalized longitudinal velocity *u*_*x*_/*u*_max_ of the particle is plotted versus time in Fig. 3e, i. Even for this physical quantity, oscillations appear around an average value, following what has been already reported for the number of active ligands. Oscillations are larger for the smaller Reynolds numbers. Indeed, for *Re* = 0.1, the average longitudinal velocity is nearly zero in the case of *ρ*_l_ = 0.7 and 0.9 and grows up to 4.08×10^−2^ for *ρ*_l_ = 0.5. For *ρ*_l_ = 0.3, the normalized longitudinal velocity is 4.80 × 10^−2^ with no oscillation in that the particle is not adhering to the wall and travels as a rigid body passively transported by the blood flow. For *Re* = 1.0, oscillations are smaller and the longitudinal velocity higher due to the larger hydrodynamic dislodging forces. Numerical values for all displacement and kinematic parameters are listed in Table 2 for ease of comparison. Table 2 shows also that for *Re* = 0.01, all particles exhibiting a ligand density *ρ*_l_ ≥ 0.5 have zero rotational and longitudinal velocity, implying that these particles can form stable bonds with the wall—*firmly adhering particles*. Differently, particles with *ρ*_l_ < 0.5 roll without adhering to the wall—*rolling*, *not adhering particles*.

### Vascular adhesion dynamics for elliptical particles

**Fig. 4:**
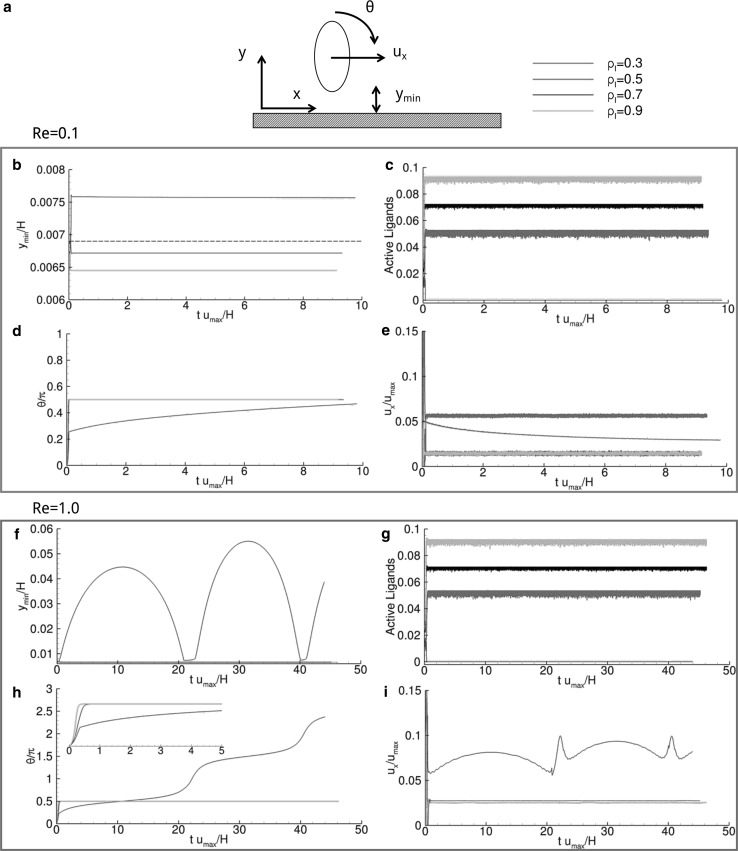
Vascular adhesion of elliptical particles ($\sigma = 2$). **a** Schematic representation of the problem. **b**, **f** Particle separation distance from the wall versus time. The dashed line corresponds to $y_{\text{cr}}$. **c**, **g** Active over total number of ligands versus time. **d**, **h** Angular rotation, $\theta,$ versus time where the inset presents a magnified view within the interval $0 \le tu_{max} \le 5$. **e**, **i** Normalized rolling velocity versus time

Data on the adhesive dynamics of elliptical particles are shown in Fig. 4 and listed in Table 3, for *Re* = 0.01, 0.1 and 1.0 and for aspect ratios equal to 2 and 3. The elliptical particle, initially settled in close proximity of the vessel wall (*y*_0_ = 3.0×10^−3^ H) and with its major axes pointing orthogonally to the wall, rapidly forms ligand-receptor bonds initiating the adhesion process (Fig. 4a). Note that the separation distance *y*_0_ is smaller than the critical distance for bond formation (*y*_cr_ = 6.8×10^−3^ H).

**Table 3:** Elliptical particle, with aspect ratio 2, kinematics and dynamics quantities obtained for *σ* = 2

Reynolds number		Ligand density			
		0.3	0.5	0.7	0.9
0.01	*y*_min_/*H*	8.2 × 10^−3^	6.1 × 10^−3^	6.1 × 10^−3^	6.1 × 10^−3^
	*u*_roll_/*u*_max_	5.00 × 10^−2^	0	0	0
	Ω*H*/*u*_max_	0.1	0	0	0
0.1	*y*_min_/*H*	7.76 × 10^−3^	6.71 × 10^−3^	6.47 × 10^−3^	6.47 × 10^−3^
	*u*_roll_/*u*_max_	2.88 × 10^−2^	5.58 × 10^−2^	1.51 × 10^−2^	1.40 × 10^−2^
	ΩH/u_max_	0.05	0	0	0
1	*y*_min_/*H*	27.3 × 10^−3^	6.71 × 10^−3^	6.47 × 10^−3^	6.47 × 10^−3^
	*u*_roll_/*u*_max_	7.81 × 10^−2^	2.75 × 10^−2^	2.52 × 10^−2^	2.52 × 10^−2^
	Ω*H*/*u*_max_	0.40	0	0	0

Separation distance, *y*_min_/*H*, rolling velocity, *u*_roll_/*u*_max_, and rotational velocity, ${\varOmega}H/u_{max}$, are tabulated as function of the Reynolds number and the density of ligands ($\rho_{l}$)

At low Reynolds number, the adhesion dynamics of elliptical particles is qualitatively similar to that of circular particles. As shown in Fig. 4b, the equilibrium position *y*_min_ is rapidly reached and preserved for the whole simulation period. At first, an abrupt variation in *y*_min_ is observed, which is related to the initial orientation of the particle with respect to the flow field and its sudden rotation. Also, as compared to circular particles, the equilibrium position *y*_min_ is slightly higher for a given ligand density *ρ*_l_. This could possibly be ascribed to higher hydrodynamic forces exerted over elliptical particles. Very differently, at *Re* = 1.0 and for sufficiently low ligand densities (*ρ*_l_ = 0.3), firm deposition of elliptical particles on the wall is impaired and the dislodging forces are strong enough to induce a periodic particle rotation over the wall—*rolling*, *not adhering particle*. This is shown in Fig. 4f where *y*_min_/H oscillates and stays constant (transient adhesion), only for a small portion of the observation time.

Furthermore, the number of closed ligand-receptor bonds is larger for elliptical particles at all given *ρ*_l_, but for *ρ*_l_ = 0.3 (Fig. 4c, g). Indeed, elliptical particles expose a larger portion of their perimeter to the wall allowing for a larger percentage of ligands to be engaged with their counter-molecules (receptors) on the wall (> twofold). Also note that, for *ρ*_l_ = 0.3, the number of closed ligand-receptor bonds is equal to zero for both circular and elliptical particles.

The angular rotation *θ* is plotted in Fig. 4d, h. For *Re* = 0.1 (Fig. 4d), particles move from the original vertical position (*θ* = 0) and progressively deposit on the wall tending to the more stable configuration *θ* = π/2. This rotation occurs quite abruptly for *ρ*_l_ larger than 0.3. Differently, for *Re* = 1.0 (Fig. 4h), the rolling and not adhering particle (*ρ*_l_ = 0.3 and *Re* = 1.0) shows a continuously growing θ with spikes in angular velocities *Ω* (local derivative of θ with respect to time) corresponding to a quasi-vertical position of the particle. For larger ligand densities, *θ* reaches the steady-state value of *θ* = π/2, implying that the particle does not rotate anymore after laying down on the wall.

Finally, the normalized longitudinal velocity *u*_*x*_/*u*_max_ is plotted in Fig. 4e, i. For all considered cases, the velocity is not zero but constant for the whole observation period besides for the rolling and not adhering particle (Fig. 4i). The not zero velocity implies that the not rotating elliptical particles, once deposited horizontally over the wall, tend to slide longitudinally breaking old bonds at the trailing edge, forming new bonds at the leading edge and along the particle body—*sliding*, *not adhering particles*. Indeed, the larger the number of active ligands, the lower the sliding velocity of the particle.

Numerical values for all displacement and kinematic parameters are listed in Table 3, for an aspect ratio 2, and Table 4, for an aspect ratio 3, for the ease of comparison. Tables 3 and 4 show also that for *Re* = 0.01, all particles exhibiting a ligand density *ρ*_l_ ≥ 0.5 have zero rotational and longitudinal velocity, implying that these particles can form stable bonds with the wall—*firmly adhering particles*. Differently, particles with *ρ*_l_ < 0.5 roll without adhering to the wall—*rolling*, *not adhering particles*.

**Table 4:** Elliptical particle, with aspect ratio 3, kinematics and dynamics quantities obtained for *σ* = 2

Reynolds number		Ligand density			
		0.3	0.5	0.7	0.9
0.01	*y*_min_/*H*	6.1 × 10^−3^	6.1 × 10^−3^	6.1 × 10^−3^	6.1 × 10^−3^
	*u*_roll_/*u*_max_	0	0	0	0
	Ω*H*/*u*_max_	0	0	0	0
0.1	*y*_min_/*H*	7.59 × 10^−3^	6.71 × 10^−3^	6.47 × 10^−3^	6.47 × 10^−3^
	*u*_roll_/*u*_max_	1.12 × 10^−2^	2.82 × 10^−2^	2.10 × 10^−2^	2.10 × 10^−2^
	Ω*H*/*u*_max_	0.04	0	0	0
1	*y*_min_/*H*	27,3 × 10^−3^	6.71 × 10^−3^	6.47 × 10^−3^	6.47 × 10^−3^
	*u*_roll_/*u*_max_	6.00 × 10^−2^	2.70 × 10^−2^	2.17 × 10^−2^	2.17 × 10^−2^
	Ω*H*/*u*_max_	0.42	0	0	0

Separation distance, *y*_min_/*H*, rolling velocity, *u*_roll_/*u*_max_, and rotational velocity, ${\varOmega}H/u_{max}$, are tabulated as function of the Reynolds number and the density of ligands ($\rho_{l}$)

### Particle–wall interaction regimens

**Fig. 5:**
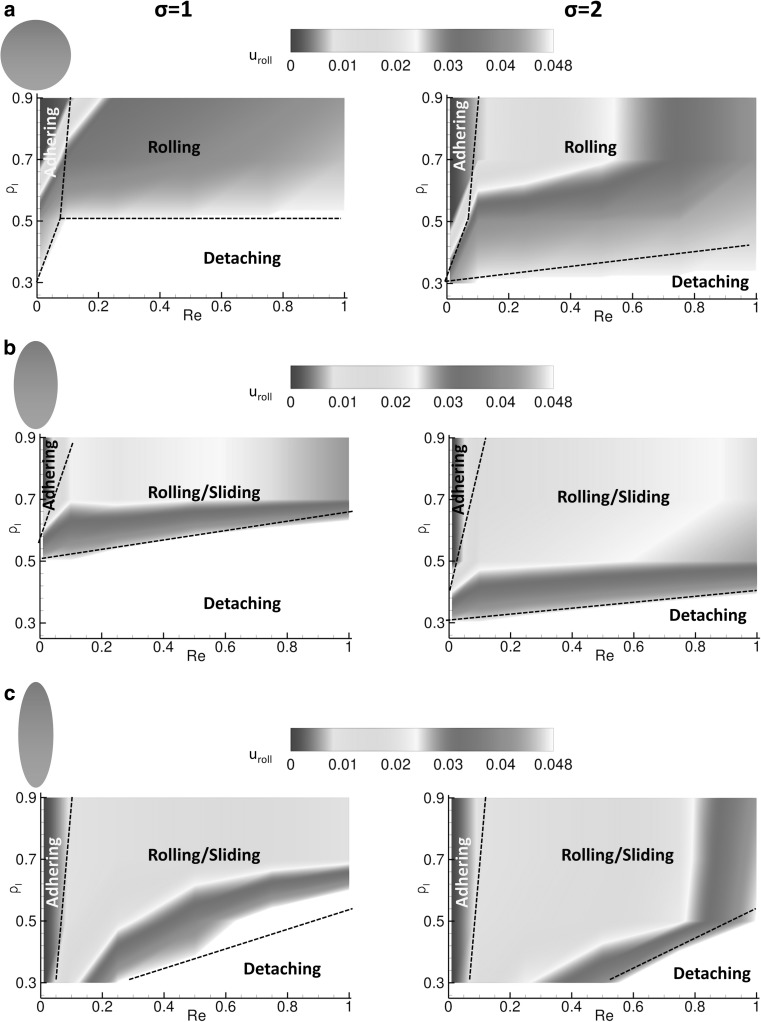
Contour plots for the rolling velocity. **a** Circular particle transport with soft ($\sigma = 1$) and rigid ($\sigma = 2$) ligand-receptor bonds. **b** Elliptical particle, with aspect ratio 2, transport with soft ($\sigma = 1$) and rigid ($\sigma = 2$) ligand-receptor bonds. **c.** Elliptical particle, with aspect ratio 3, transport with soft ($\sigma = 1$) and rigid ($\sigma = 2$) ligand-receptor bonds

**Fig. 6:**
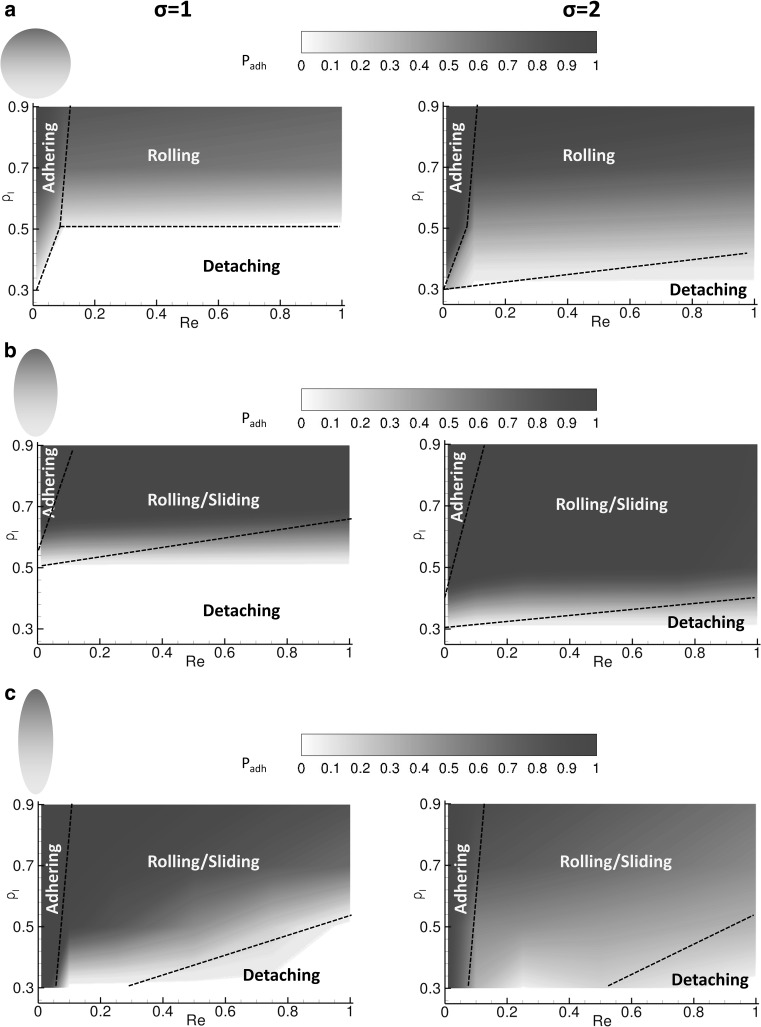
Contour plots for the probability of adhesion. **a** Circular particle transport with soft ($\sigma = 1$) and rigid ($\sigma = 2$) ligand-receptor bonds. **b** Elliptical particle, with aspect ratio 2, transport with soft ($\sigma = 1$) and rigid ($\sigma = 2$) ligand-receptor bonds. **c** Elliptical particle, with aspect ratio 3, transport with soft ($\sigma = 1$) and rigid ($\sigma = 2$) ligand-receptor bonds

**Fig. 7:**
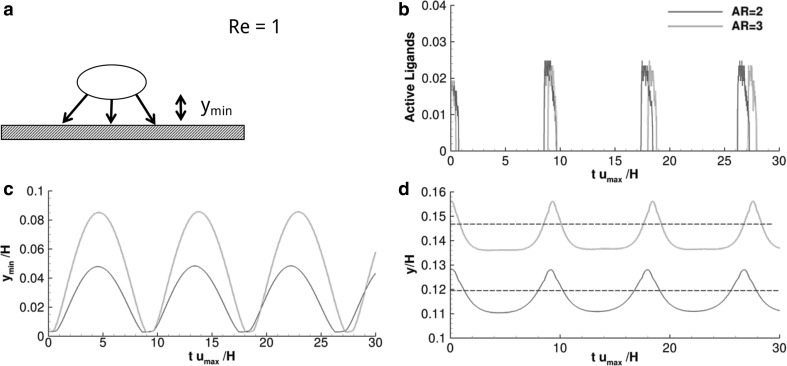
Vascular transport of elliptical particles with different critical bond length. **a** Schematic representation of the problem. **b** Active over total number of ligands versus time. **c** Particle separation distance from the wall versus time. **d** Centroid lateral position versus time

As described in the previous paragraphs, depending on the flow and particle properties, different regimens of particle–wall interaction can be documented: firmly adhering, rolling, sliding and not adhering particles. This is summarized in Figs. 5 and 6, where the rolling velocity *u*_roll_/*u*_max_ and probability of adhesion *P*_a_ are presented as a function of the considered three different shapes—circular and elliptical with aspect ratios 2 and 3, ligand densities *ρ*_l_—ranging from 0.3 to 0.9, Reynolds numbers—varying from 0.01 to 1.0, and bond strength *σ*—equal to 1 (soft bond) and 2 (rigid bond). Figure 5 presents a contour plot for the normalized rolling velocity *u*_roll_/*u*_max_, whereas Fig. 6 gives a contour plot for the probability of adhesion, *P*_a_. This quantity is defined as the ratio between the number of active bonds and the maximum number of bonds that can be closed at any given time during the adhesion process and represents the likelihood of forming stable bonds at the particle–wall interface. The maximum number of bonds is readily calculated as a function of the particles geometry and orientation with respect to the wall. Both physical quantities ($u_{r}$ and $P_{\text{a}}$) are affected in a similar fashion by the flow and particle properties. Specifically, low Reynolds numbers and high ligand densities (upper-left area) are associated with zero rolling velocities and *firmly adhering particles*. Indeed, under these conditions, the hydrodynamic dislodging forces are moderately low (low Re) and are readily balanced by the high adhesive interactions (high *ρ*_l_). At the other extreme, high Reynolds numbers and low ligand densities (lower-right area) are associated with *not adhering particles*. Under these conditions, the hydrodynamic dislodging forces (high *Re*) cannot be balanced by the adhesive interactions (low *ρ*_l_). In between these two limiting conditions, particles are observed to move relatively to the substrate. With circular particles and elliptical particles at moderate *ρ*_l_, continuous rolling over the wall is documented. On the other hand, with elliptical particles at high *ρ*_l_, longitudinal sliding over the wall is observed. Note that rolling of elliptical particles is limited by their larger rotational inertia. However, longer bonds may facilitate rolling and slender particles as depicted in Fig. 7. Finally, adhesion is favored by stronger bonds in that, for fixed dislodging forces, higher σ are associated with lower ligand-receptor bond energies (∝ $F_{b}^{2}$/*σ*).

## Conclusions and future perspectives

A combined lattice Boltzmann–immersed boundary (LB–IB) model was developed for predicting the adhesive interactions of circulating particles with walls lining a blood vessel. Particles were decorated with ligand molecules forming molecular bonds with counter-molecules (receptors) uniformly distributed over the wall. Three different particle shapes were considered (circular and elliptical with aspect ratios 2 and 3) and transported in a linear laminar flow, characterized by physiologically relevant Reynolds numbers (from 0.01 to 1.0).

First, the computational model was validated by estimating the velocities of quasi-circular cells rolling over a continuous endothelial layer in a microfluidic chip. For different values of the Reynolds number, predictions from the LB–IB model were in good agreement with experimental data, thus confirming the accuracy of the proposed approach. Then, the interaction of circular and elliptical particles with the wall was studied varying systematically the particle shape, ligand density, ligand-receptor bond strength and flow conditions. As a function of the above independent parameters, particle–wall interaction maps were derived documenting four possible regimens: firmly adhering, sliding, rolling and not adhering particles.

The proposed LB–IB model can be accurately employed to predict the vascular dynamics and adhesion interactions of systemically injected particles. Relevant biophysical parameters can be efficiently modulated allowing for systematic analyses and supporting the rational design of particles for drug delivery and imaging.

## Electronic supplementary material

Below is the link to the electronic supplementary material.

Bright field microscopy movie showing HCT-15 cancer cells flowing (*Q* = 100 nL/min) into a PDMS microfluidic chip over a confluent monolayer of HUVECs (× 10 magnification, scale bar 250 μm). A few individual cells are observed to transiently adhere and roll over the HUVEC monolayer, at the bottom of the microfluidic chip. Other cells, debris, and a few cell clusters are clearly observed to move downstream transported by the flow, without interacting with the HUVEC monolayer at the bottom (MP4 46932 kb)
